# Nitrogen-Doped Carbon Nanoparticles Derived from Silkworm Excrement as On–Off–On Fluorescent Sensors to Detect Fe(III) and Biothiols

**DOI:** 10.3390/nano8060443

**Published:** 2018-06-17

**Authors:** Xingchang Lu, Chen Liu, Zhimin Wang, Junyi Yang, Mengjing Xu, Jun Dong, Ping Wang, Jiangjiang Gu, Feifei Cao

**Affiliations:** College of Science, Huazhong Agricultural University, Wuhan 430070, China; xingchanglu0514@163.com (X.L.); 18110457321@163.com (C.L.); 15071367153@163.com (Z.W.); 13437163425@163.com (J.Y.); 15858203295@163.com (M.X.); 15271945456@163.com (J.D.); Wang5wp@sina.com (P.W.)

**Keywords:** nitrogen-doped carbon nanoparticles, silkworm excrement, on–off–on fluorescent sensors, Fe(III), biothiols

## Abstract

On–off–on fluorescent sensors based on emerging carbon nanoparticles (CNPs) or carbon dots (CDs) have attracted extensive attention for their convenience and efficiency. In this study, dumped silkworm excrement was used as a novel precursor to prepare fluorescent nitrogen-doped CNPs (N-CNPs) through hydrothermal treatment. The obtained N-CNPs showed good photoluminescent properties and excellent water dispersibility. Thus, they were applied as fluorescence “on–off–on” probes for the detection of Fe(III) and biothiols. The “on–off” process was achieved by adding Fe(III) into N-CNP solution, which resulted in the selective fluorescence quenching, with the detection limit of 0.20 μM in the linear range of 1–500 μM. Following this, the introduction of biothiols could recover the fluorescence efficiently, in order to realize the “off–on” process. By using glutathione (GSH) as the representative, the linear range was in the range of 1–1000 μM, and the limit of detection was 0.13 μM. Moreover, this useful strategy was successfully applied for the determination of amounts of GSH in fetal calf serum samples.

## 1. Introduction

Biothiols, such as glutathione (GSH), cysteine (Cys), and homocysteine (Hcy) are small molecular weight biomolecules with thiol groups, which play crucial roles in numerous biological processes [1,2,3]. For example, GSH is abundant in cells to help maintain the cellular redox homeostasis, control signal transduction, and take part in gene regulation [4,5]. The abnormal concentration of biothiols is related to various ailments and disorders, including AIDS, HIV, Alzheimer’s disease, liver damage, heart disease, and cancer [6,7,8]. Moreover, biothiols are also widely used in the food industry, as well as in cosmetic and pharmaceutical areas, due to their unique physiological properties [9]. Consequently, the analysis of biothiols in biological or environmental systems is of great importance. Although many analytical strategies, including colorimetry [10], high performance liquid chromatography (HPLC) [11], electrochemistry [12], and surface-enhanced Raman scattering (SERS) [13] have been developed for the detection of biothiols, the fluorescent method is thought of as a powerful kind of technology because of its easy operation, high sensitivity, and flexibility [14,15]. In recent years, several fluorescent systems based on emerging carbon nanoparticles (CNPs) or carbon dots (CDs) have been developed for sensing biothiols. Most of these strategies are based on the fluorescence “on–off–on” mechanism, which needs an intermediate to quench the photoluminescence, and biothiols to recover the fluorescence. However, almost all intermediates are heavy metal ions such as Hg(II) [16,17,18,19], Ag(I) [20,21], Cu(II) [22,23], and Au(III) [24], which are not eco-friendly, and will cause serious problems to the environment and public health. Thus, searching for an alternative ion (e.g., Fe^3+^) is extremely important for this kind of detection platform, which is application-oriented. In addition, Fe(III) detection is of vital importance, since Fe(III) is essential for many biological systems, and a deficiency of Fe(III) could cause various diseases, including anemia, renal failure, and liver injury [25,26,27].

Fluorescent CNPs and CDs are novel carbon-based nanomaterials with good biocompatibility, eco-friendliness, excellent photoluminescence, and high photostability, and are widely applied in chemical sensing, bio-imaging, and other fields [28,29,30,31,32]. The synthetic approaches of CNPs and CDs are usually classified as top-down cutting of bulk carbon sources (graphite [33,34], graphene [35], carbon nanotubes [36], coal [37] etc.) and bottom-up carbonization of molecules (citric acid [38,39], glucose [40], phenylenediamine [41,42] etc.). It is believed that the raw materials are related to the properties of prepared CNPs or CDs, including fluorescent emission and solubility [41,43]. Biomass is abundant in nature, and supplies quantities of carbon and heteroatoms, which will participate in the synthetic procedure, and help passivate the product itself. Therefore, the employment of bio-resources is becoming more and more popular, and is considered to be a low-cost and renewable route to obtaining low biotoxic CNPs and CDs [44,45,46,47]. Silkworm excrement is an agricultural waste produced by *Bombyx mori* in quantities of millions of metric tons every year, and has always been utilized as a traditional Chinese medicine and fertilizer [48,49]. In fact, silkworm excrement is mainly composed of fibers, fats, proteins, amino acids and so on, which is suitable for the preparation of nitrogen-doped CNPs.

In this paper, novel nitrogen-doped CNPs (N-CNPs) were prepared by the hydrothermal treatment of silkworm excrement that could be employed as both a carbon and a nitrogen source. These as-prepared N-CNPs showed bright blue emission under ultraviolet illumination, with excellent aqueous solubility. The strong fluorescence of N-CNPs could be selectively quenched by Fe(III) to form an N-CNPs/Fe(III) system. This quenched system could be restored by adding biothiols. Thus, the fluorescence “on–off–on” process was achieved for the detection of Fe(III) and biothiols. Furthermore, the GSH in the practical sample was successfully evaluated.

## 2. Materials and Methods

### 2.1. Materials and Chemicals

Silkworm excrement was obtained from sericulturists (Zhejiang, China) and washed to remove impurities. AgNO_3_, AlCl_3_·6H_2_O, CaCl_2_, CdCl_2_·2.5H_2_O, CrCl_3_·6H_2_O, FeCl_2_·4H_2_O, FeCl_3_·6H_2_O, KCl, LiCl, MnCl_2_·4H_2_O, NaCl, NH_4_Cl, NiCl_2_·6H_2_O, PbCl_2_, ZnCl_2_ were provided by Sinopharm Chemical Reagent Co., Ltd. (Shanghai, China). Glucose, α-Lactose, sucrose, dopamine hydrochloride, β-alanine, l-arginine, l-asparagine, l-glutamic acid, glycine, l-histidine, l-methionine, l-phenylalanine, l-proline, dl-homocysteine, l-cysteine, reduced l-glutathione of analytical reagent grade were purchased from Aladin Ltd. (Shanghai, China). The fetal calf serum (main compositions: globular protein and bovine serum albumin) was obtained from Tianhang Biotechnology Co., Ltd. (Zhejiang, China). Ultrapure water (18.2 MΩ cm^−1^) obtained from the Millipore system was used in this study.

### 2.2. Characterization

Transmission electron microscopy (TEM) images were observed by a H-7650 electron microscope (Hitachi, Tokyo, Japan) at an accelerating voltage of 80 kV. The N-CNPs were dropped onto 400-mesh carbon-coated Cu grids and dried at room temperature. X-ray diffraction (XRD) patterns were obtained by using a D8 Advance X-ray diffractometer (Bruker, Billerica, MA, USA) with a filtered Cu Ka radiation. Fourier transform infrared (FT-IR) spectroscopy was performed on a NEXUS 670 FTIR spectrometer (Thermo Scientific, Waltham, MA, USA), ranging from 400–4000 cm^−1^. The N-CNPs were dispersed in KBr pellets. X-ray photoelectron spectroscopy (XPS) results were collected using an ESCALab 250Xi XPS instrument (Thermo Scientific, Waltham, MA, USA). Ultraviolet-visible (UV-vis) absorption spectrum was recorded by a Shimadzu 2450 UV-vis spectrophotometer (Shimadzu, Kyoto, Japan). All fluorescence spectra were conducted by a RF-5301PC fluorescence spectrometer (Shimadzu, Kyoto, Japan) with 5 nm slit width for the excitation and 10 nm slit width for the emission.

### 2.3. Preparation and Purification of Nitrogen-Doped CNPs (N-CNPs)

150 mg of silkworm excrement and 10 mL of ultrapure water were added into 15 mL of Teflon equipped stainless steel autoclave. The mixture was heated at 200 °C for 24 h and cooled down to room temperature naturally. The obtained yellow solution was centrifuged at 10,000 rpm for 15 min to precipitate out insoluble precipitate, and filtered by 220 nm membrane. Following this, the solution was dialyzed for 1 day (MWCO = 3.5 kD). Finally, the product of N-CNPs was collected by freeze-drying for further use.

### 2.4. Selectivity and Quantitative Measurements for Fe(III) and Biothiols

The detection of Fe(III) and biothiols was performed at pH = 7.0, and at room temperature. For Fe(III) detection, 400 μL of N-CNPs (0.1 mg mL^−1^, pH = 7.0) was dispersed into 3.2 mL of Tris-HCl buffer (pH = 7.0), then 400 μL of Fe(III) solution (pH = 7.0) was added, and the final concentration of Fe(III) was in the range of 0–500 μM. For comparison, the same procedure was performed for other ions at the final concentration of 500 μM. To detect biothiols, GSH was chosen and added into an N-CNPs/Fe(III) complex solution with different concentrations (0, 1, 2, 5, 10, 20, 50, 100, 200, 400, 600, 800, and 1000 μM). The same procedure was also performed for other biomolecules at the final concentration of 1 mM. All the emission spectra were recorded at 340 nm excitation.

### 2.5. The Detection of Glutathione (GSH) in Fetal Calf Serum Samples

The fetal calf serum was used to evaluate the detection ability of N-GNPs/Fe(III) in a practical situation. All the samples were filtered through 0.22 μm membranes to remove the large suspended particles, and their pH levels were regulated to 7.0. Then, 2.8 mL of Tris-HCl, 400 μL of sample, 400 μL of Fe(III) solution, and 400 μL of N-CNPs were mixed and recorded with the fluorescence spectrometer.

### 2.6. Quantum Yield (QY) Measurement

The QY of N-CNPs was calculated by the equation below as Equation (1):

QY_s_ = QY_r_·*I*_s_*A*_r_*η*_s_^2^/(*I*_r_*A*_s_*η*_r_^2^) (1)

where “s” and “r” refer to the sample and reference, respectively. The reference quinine sulfate is dissolved in 0.1 M H_2_SO_4_ and its QY is 0.54 at 340 nm [50]. “*I*” represents the integrated emission intensity of fluorescent spectra at the excited wavelength of 340 nm. “*A*” is the UV-vis absorbance at 340 nm, controlled as 0.1–0.01 to avoid re-absorption. “*η*” is the refractive index of the solvent.

## 3. Results and Discussion

**Figure 1:**
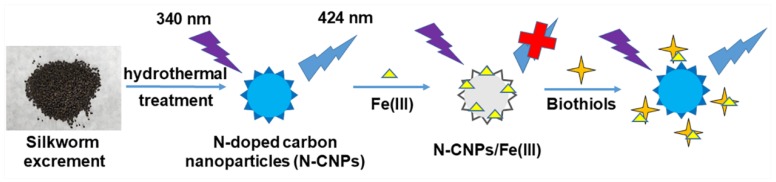
A schematic illustration of the formation of nitrogen-doped CNPs (N-CNPs) from silkworm excrement, and the fluorescence “on–off–on” detection of Fe(III) and biothiols.

The synthesis of N-CNPs from silkworm excrement and the fluorescence “on–off–on” strategy to detect Fe(III) and biothiols were summarized in Figure 1. The silkworm excrement was a deep gray granulum which could not be dispersed in water. However, under the high temperature and pressure of the hydrothermal process, the silkworm excrement was likely decomposed, and carbonized to form N-CNPs with self-passivation. The N-CNPs were stable, and demonstrated good blue photoluminescence under UV excitation, which could be quenched in the presence of Fe(III), and formed the N-CNPs/Fe(III) mixture. In a further step, the weak fluorescence of N-CNPs/Fe(III) could be restored by biothiols though the interaction between Fe(III) and biothiols.

**Figure 2:**
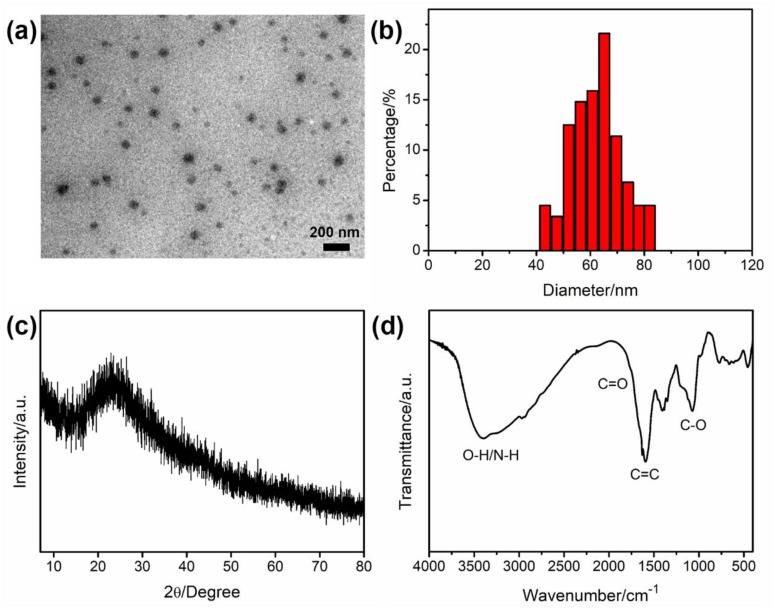
Transmission electron microscopy (TEM) image (**a**); the particle size distribution histogram (**b**); X-ray diffraction (XRD) pattern (**c**); and fourier transform infrared (FT-IR) spectrum (**d**) of N-CNPs.

TEM was employed to characterize the morphology and size distribution of N-CNPs. As demonstrated in Figure 2a, the N-CNPs were well-separated spherical nanoparticles. The corresponding particle size distribution histogram in Figure 2b showed that the N-CNPs ranged from 40–85 nm in size, and the mean diameter was about 62 nm. Although N-CNPs had a larger size than traditional CDs (usually less than 10 nm), their solution was stable for several weeks without any aggregate and precipitate.

To confirm the crystallinity of N-CNPs, XRD was measured, and the result was shown in Figure 2c. It was clear that a broad diffraction peak (2θ) was observed at 23.3°, which revealed that N-CNPs had an amorphous crystal phase [51]. FT-IR was used to identify the functional groups and chemical structure of N-CNPs. As shown in Figure 2d, the peaks at 3391 and 3247 cm^−1^ were attributed to O–H/N–H stretching vibration. The intense peak at around 1586 cm^−1^ was attributed to the vibration of C=C, which was formed in the hydrothermal process. The small absorption peak at 1752 cm^−1^ indicated the C=O group. The other peak at 1071 cm^−1^ was assigned to the C–O and C–O–C bands.

**Figure 3:**
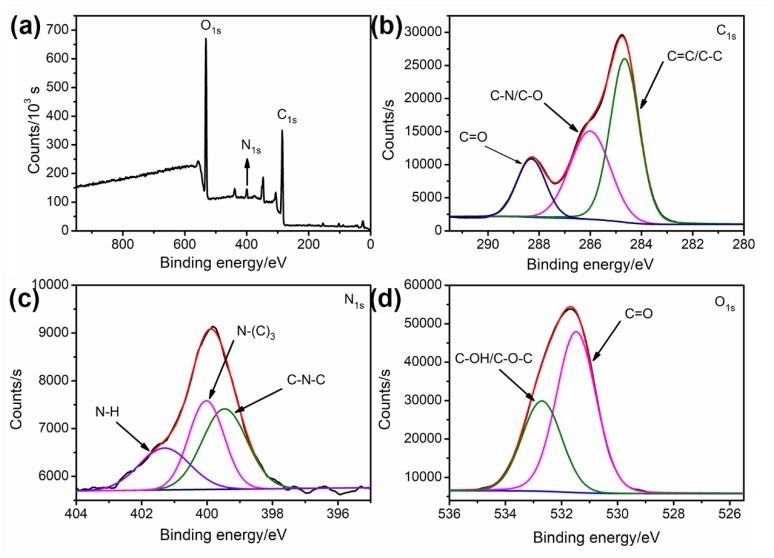
X-ray photoelectron spectroscopy (XPS) full survey (**a**); C 1s (**b**); N 1s (**c**) and O 1s (**d**) spectra of N-CNPs. Black line: raw, navy line: background, other lines: fitting.

XPS analysis was used to investigate the surface composition and element states of the N-CNPs. The three peaks centered at 285.0, 399.1, and 532.0 eV in the XPS spectra of N-CNPs (Figure 3a) were ascribed to C 1s, N 1s, and O 1s, which had an atomic ratio of 61.51:3.99:34.5. The presence of the N 1s peak revealed that the nitrogen was successfully doped in the resultant CNPs. Furthermore, the C 1s spectrum in Figure 3b had three peaks at 284.7, 286.1, and 288.3 eV, which were assigned to C=C/C–C, C–N/C–O, and C=O groups, respectively. The three peaks at 399.5, 400.0, and 401.3 eV in the N 1s spectrum (Figure 3c) were attributed to C–N–C, N–(C)_3_, and N–H respectively. The O 1s spectrum in Figure 3d was deconvoluted into two peaks at 531.5 and 532.7 eV, which were attributed to C=O and C–OH/C–O–C, respectively. The existence of various functional groups in N-CNPs created a good dispersibility in the aqueous solution system.

**Figure 4:**
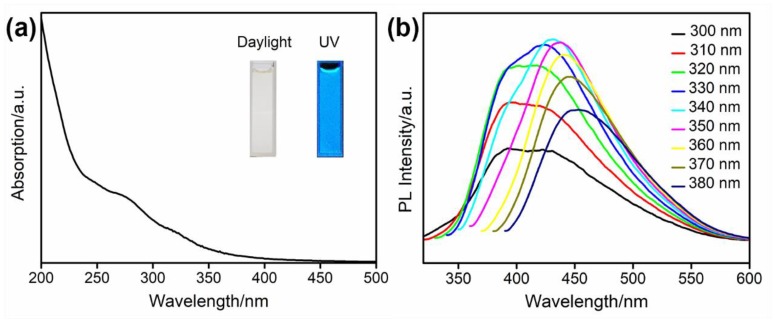
Ultraviolet-visible (UV-vis) absorption spectrum (**a**) and photoluminescent (PL) emission spectra at different excitation wavelengths (**b**) of N-CNPs. Inset: photographs of N-CNPs in water under daylight and UV irradiation.

The UV-vis absorption and photoluminescent (PL) emission spectra were explored to investigate the optical properties of N-CNPs. As shown in Figure 4a, the UV-vis absorption spectrum of N-CNPs had two peaks at about 274 and 318 nm, which were caused by π → π* transition of the conjugate structure, and n → π* transition of the C=O bond [52,53]. The inset photographs in Figure 4a display N-CNPs dispersed in water under daylight (left), and under UV illumination (right). It was evident that N-CNPs emitted bright blue fluorescence under 365 nm excitation. Furthermore, the PL emission spectra were recorded under various excitation wavelengths, and exhibited in Figure 4b. The wavelength of the emission peak shifted from 392 to 451 nm when the excitation wavelength increased from 300 to 380 nm. The excitation-dependent redshift of the emission was consistent with previous CNPs or CDs, which probably resulted from the difference in size of the carbon cores, and the complex surface defects [54,55,56]. The emission peak reached its maximum at 424 nm under 340 nm excitation, which was set as the detective condition. The QY of the prepared N-CNPs was calculated as 13.1% in Table S1, with quinine sulfate as the standard reference. It was found that N-CNPs had good PL properties, making them suitable for further analytical application.

The influence of pH (1–13) on the emission spectra of N-CNPs was researched, and the PL intensity of 424 nm under 340 nm excitation was recorded in Figure S1. With the increase in pH, the PL intensity increased at first and then decreased. The N-CNPs performed a high PL intensity in neutral conditions, and the strong acid and alkaline environment would reduce the fluorescence intensity of N-CNPs, possibly due to the protonation and deprotonation effect [16]. Thus the pH = 7.0 was chosen as the optimal detection condition for the following experiment.

**Figure 5:**
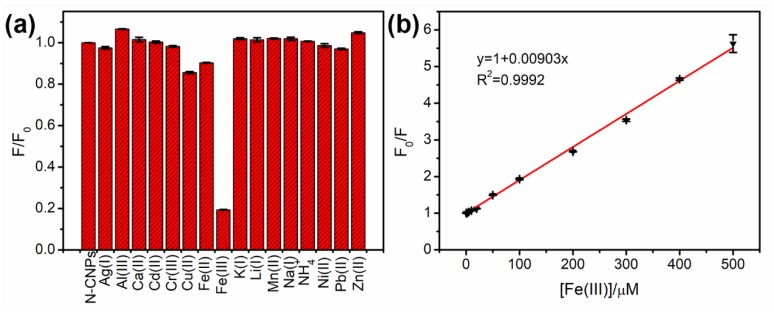
(**a**) Selectivity of N-CNPs with different ions at the same concentration (500 μM); (**b**) The linear relationship between F_0_/F and Fe(III) concentration. F and F_0_ were the PL intensities (424 nm) of N-CNPs at 340 nm excitation in the presence and absence of ions, respectively.

To explore the specific ability of N-CNPs towards Fe(III), the fluorescence emissions of N-CNPs with various positive ions (500 μM) were measured at 340 nm excitation and exhibited in Figure S2. It was clear that the emission peak of N-CNPs decreased remarkably with the addition of Fe(III). The relationship between F/F_0_ (the ratio of PL intensities of N-CNPs in the presence and absence of ions) and ion species is shown in Figure 5a. Most of the added ions including Ag(I), Al(III), Ca(II), Cd(II), Cr(III), K(I), Li(I), Mn(II), Na(I), NH_4_^+^, Ni(II), Pb(II), and Zn(II) did not cause an obvious fluorescence change of the N-CNPs, while Cu(II) and Fe(II) could partially quench the fluorescence of N-CNPs. N-CNPs with a Fe(III) system had the lowest value compared with N-CNPs with other ions. The corresponding quenching efficiency was over 80%, which confirmed that N-CNPs had a selective response to Fe(III).

Figure S3 exhibited the fluorescence spectra of N-CNPs with different concentrations of Fe(III). It was clear that the PL intensity of 424 nm decreased gradually, while further increasing the Fe(III) concentration from 0 to 500 μM. The relationship between F_0_/F (the ratio of PL intensities of N-CNPs in the absence and presence of Fe(III)) and [Fe(III)] (the concentration of Fe(III)) was presented in Figure 5b. A good fitted line (R^2^ = 0.9992) was observed and the equation was shown as Equation (2):

F_0_/F = 1 + 0.00903[Fe(III)] (2)

The LOD (limit of detection) was calculated as 0.20 μM (S/N = 3, S/N: the signal to noise ratio, based on the standard deviation of 10 blank measurements) [57]. The performance of N-CNPs was compared with the recently reported CNPs or CDs (Table S2) and exhibited lower LOD, wider linear range, or better selectivity. These results indicated that the N-CNPs could be applied as a fluorescent probe to detect Fe(III) quantitatively in the solution. It should also be noted that the limit of quantification (LOQ) was 0.66 μM according to S/N = 10 [58].

The mechanism of the selective fluorescence quenching effect of N-CNPs with Fe(III) was possibly related to the high affinity of Fe(III) towards O/N-containing groups on the surface of N-CNPs, which led to the formation of the N-CNPs/Fe(III) complex [59]. The UV-vis absorption spectrum of N-CNPs with Fe(III) was further investigated, and showed a new absorption peak at 290 nm (Figure S4), indicating the stable metal ion/N-CNP structure was formed to realize the static quenching effect [60,61].

**Figure 6:**
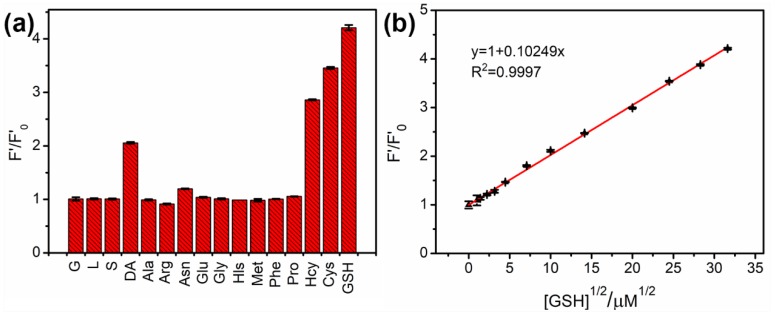
(**a**) Selectivity of N-CNPs/Fe(III) to different biological molecules at the same concentration (1 mM); (**b**) The linear relationship between (F’/F’_0_) and the square root of glutathione (GSH) concentration. F’ and F’_0_ were the PL intensities (424 nm) of N-CNPs/Fe(III) at 340 nm excitation in the absence and presence of biological molecules, respectively.

Since Fe (III) also has a great chelation towards biothiols, their strong coordination could recover the quenched PL emission of the N-CNPs/Fe(III) system. To confirm the fluorescent restoration of biothiols, various biological molecules (1 mM) including glucose (G), sacrose (S), lactose (L), dopamine (DA), alanine(Ala), arginine(Arg), asparagine(Asn), glutamic acid(Glu), glycine (Gly), histidine (Hls), methionine(Met), phenylalanine (Phe), proline (Pro), homocysteine(Hcy), cysteine (Cys), and glutathione (GSH) were added into N-CNPs/Fe(III) (N-CNPs with 500 μM Fe(III)). The fluorescence spectra were recorded at 340 nm excitation, and shown in Figure S5. The relationship between F’/F’_0_ (the ratio of PL intensities of N-CNPs/Fe(III) in the presence and absence of biological molecules) and biomolecule species was shown in Figure 6a. Saccharides and non-thiol amino acids did not have a significant influence on fluorescence intensities, while dopamine (DA) could partially restore the fluorescence of N-CNPs/Fe(III). Biothiols could dramatically enhance the weak fluorescence of N-CNPs/Fe(III), probably due to their interaction with Fe(III), and GSH performed the highest F’/F’_0_ of 4.21 ± 0.05 compared with Hcy (2.86 ± 0.01) and Cys (3.45 ± 0.02). This result confirmed the specificity of the sensing system towards biothiols. Then, we chose GSH as the typical analyte, and the enhancement was dependent on the GSH concentration. As shown in Figure S6, the fluorescent intensity increased with the increase in concentration of GSH, which revealed that the Fe(III) could be separated from N-CNPs. The F’/F’_0_ and [GSH]^1/2^ had a good fitted line (R^2^ = 0.9997) as shown in Figure 6b, which could be summarized as Equation (3):

F’/F’_0_ = 1 + 0.10249[GSH]^1/2^ (3)

The LOD was 0.13 μM with S/N = 3, which was equal to or lower than other reported fluorescent sensors based on CNPs or CDs (Table S3).

To evaluate the detection performance of N-CNPs/Fe(III) towards GSH in a complicated biological environment, serum sample was tested, and the results are shown in Table 1. Different spiked concentrations of GSH were added into the fetal calf serum samples with N-CNPs. The recovery efficiencies (the ratio of found GSH and added GSH) were 108.7, 103.9, and 102.4% for 4, 50, and 100 μM GSH, respectively. The corresponding relative standard deviations (RSD) are 1.50, 2.14, and 2.43%, respectively. These results revealed that the N-CNPs could be used for the determination of GSH in practical samples.

## 4. Conclusions

In summary, a facile and one-step hydrothermal process was used to prepare N-CNPs by applying silkworm excrement as the suitable nitrogen and carbon source. The obtained N-CNPs could be used as efficient fluorescence “on–off–on” probes to detect Fe(III) and biothiols. Fe(III) could quench the fluorescence of N-CNPs selectively to form N-CNPs/Fe(III), and the linear range was 1–500 μM with the LOD of 0.20 μM. Upon the addition of biothiols, the N-CNPs/Fe(III) could be restored efficiently. By using GSH as the example, the linear range was 1–1000 μM, with the LOD of 0.13 μM. Additionally, the platform was successfully used to evaluate the amount of GSH in a fetal calf serum sample, which achieved the goal for practical use. This novel detection strategy will help in the design of biological and environmental sensors based on fluorescent carbon nanoparticles.

## Figures and Tables

**Table 1:** Nitrogen-doped CNPs (N-CNPs) for the detection of glutathione (GSH) in fetal calf serum samples.

Added GSH (μM)	Found GSH (μM)	Recovery (%)	RSD (%)
4	4.35	108.7	1.50
50	51.96	103.9	2.14
100	102.4	102.4	2.43

## Supplementary Materials

The following are available online at http://www.mdpi.com/2079-4991/8/6/443/s1, Figure S1: pH effect on PL intensity (424 nm) of N-CNPs under 340 nm excitation, Figure S2: PL emission spectra of N-CNPs in absence and presence of various ions under 340 nm excitation, Figure S3: PL emission spectra of N-CNPs with different concentrations of Fe(III), Figure S4: UV-vis absorption spectra of N-CNPs/Fe(III) and N-CNPs, Figure S5: PL emission spectra of N-CNPs/Fe(III) in absence and presence of different biomolecules under 340 nm excitation, Figure S6: PL emission spectra of N-CNPs/Fe(III) with different concentrations of GSH, Table S1: QY calculation of N-CNPs, Table S2: The comparison between various CNPs and CDs toward Fe(III), Table S3: The comparison between various fluorescent sensors based on CNPs or CDs toward GSH.
