# Supplementary material for: Nitrogen-Doped Carbon Nanoparticles Derived from Silkworm Excrement as On–Off–On Fluorescent Sensors to Detect Fe(III) and Biothiols
Source: Nanomaterials (Basel). 2018 Jun 17;8(6):443. doi: 10.3390/nano8060443 (PMC6027355; doi:10.3390/nano8060443)
Supplement: Supplementary file 1 [file nanomaterials-08-00443-s001.pdf]

Nitrogen-doped Carbon Nanoparticles Derived from Silkworm Excrement as On-off-on Fluorescent Sensors to Detect Fe(III) and Biothiols

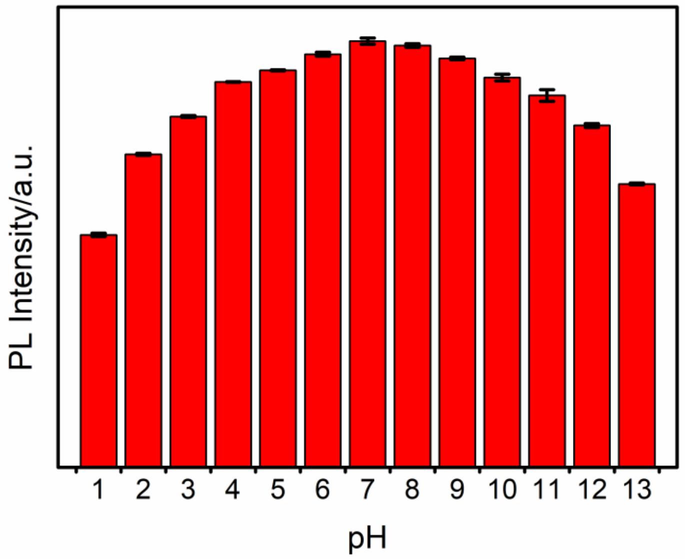

Figure S1. pH effect on PL intensity (424 nm) of N-CNPs under 340 nm excitation.

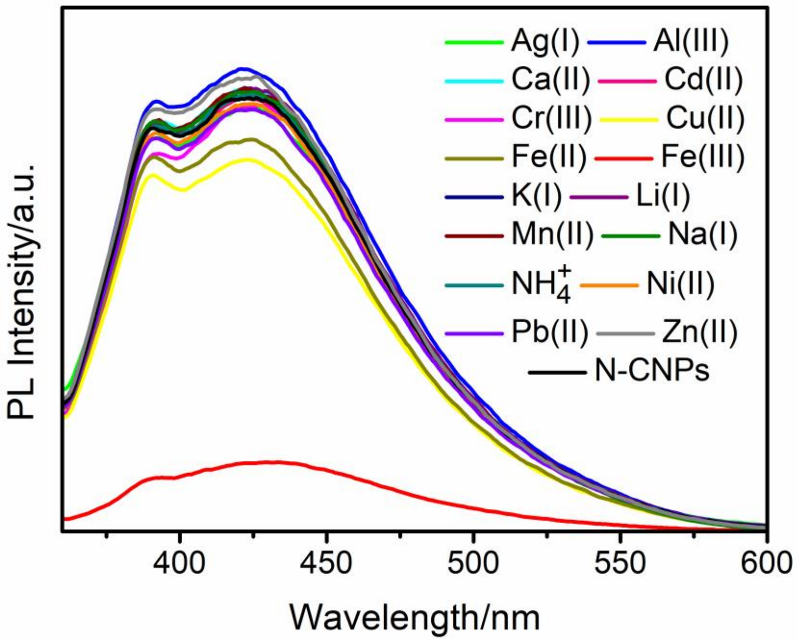

Figure S2. PL emission spectra of N-CNPs in absence and presence of various ions under 340 nm excitation.

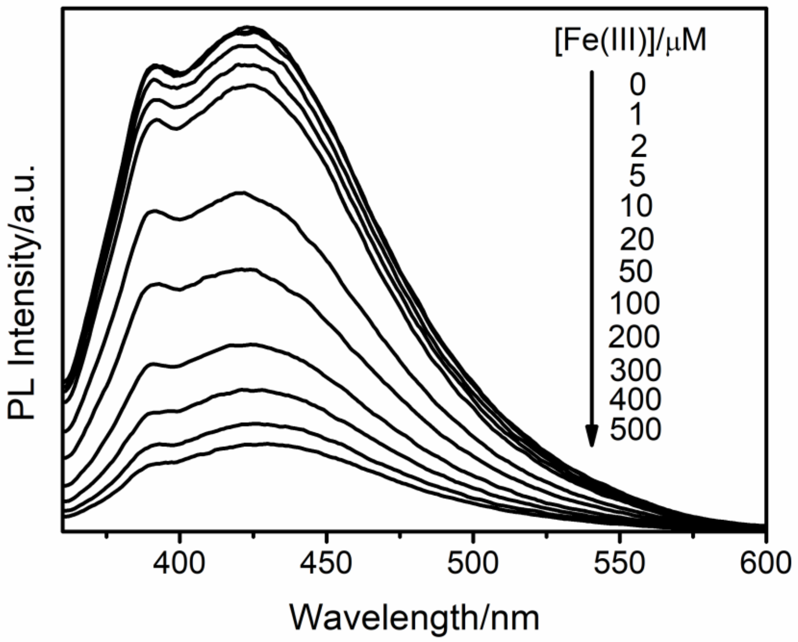

Figure S3. PL emission spectra of N-CNPs with different concentrations of Fe(III).

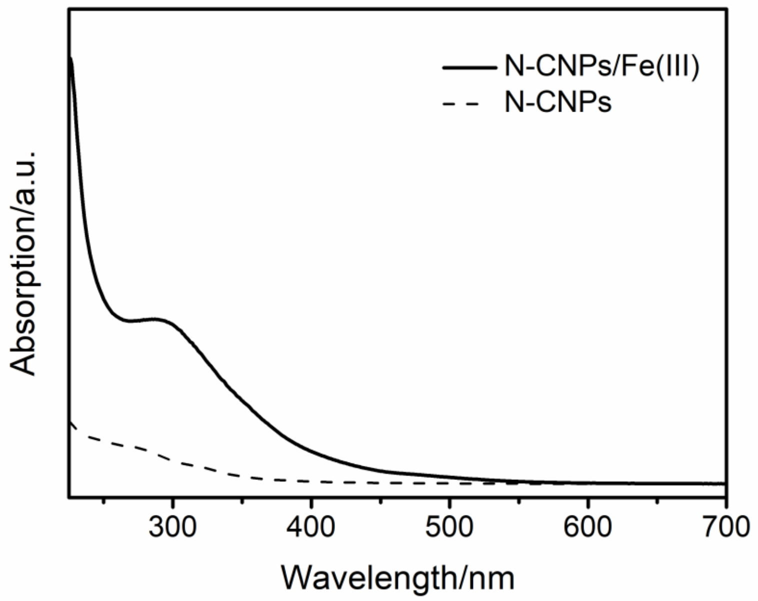

Figure S4. UV-vis absorption spectra of N-CNPs/Fe(III) and N-CNPs.

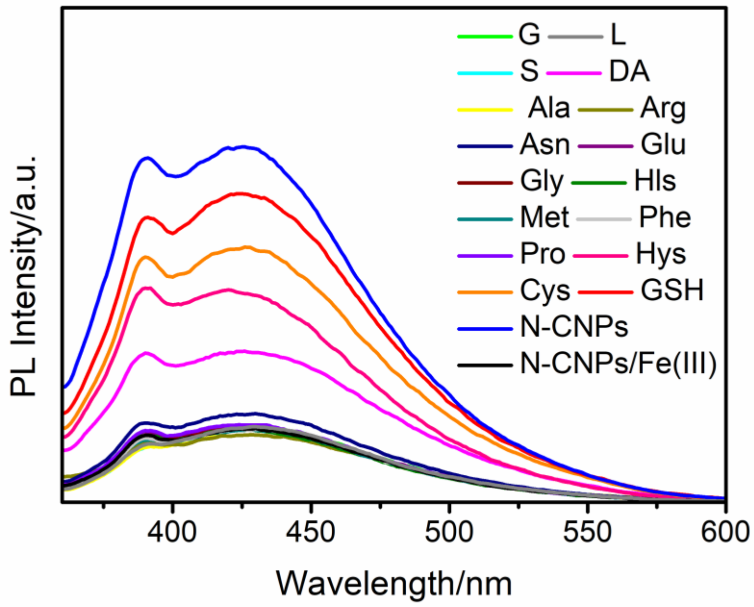

Figure S5. PL emission spectra of N-CNPs/Fe(III) in absence and presence of different biomolecules under 340 nm excitation.

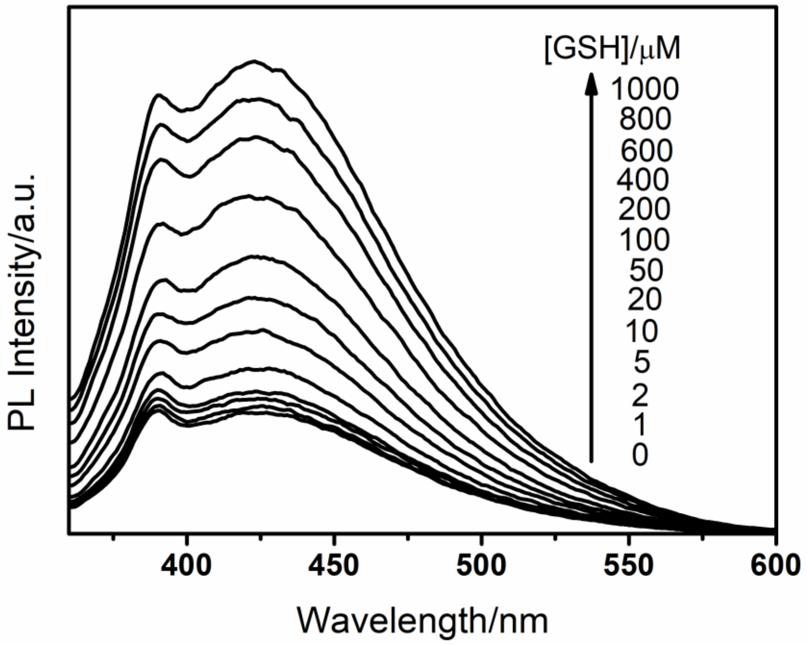

Figure S6. PL emission spectra of N-CNPs/Fe(III) with different concentrations of GSH.

Table S1. QY calculation of N-CNPs.

Sample	I	A	η	QY
Quinine sulfate	25533.83	0.015	1.333	0.54
N-CNPs	29656.837	0.072	1.333	0.131

Table S2. The comparison between various CNPs and CDs toward Fe(III).

Material	LOD (μM)	Linear range (μM)	Response towards metal ions	Reference
N-CDs	0.5	0–150	Fe(III), Cr(VI)	[S1]
CDs	0.239	0–80	Fe(III)	[S2]
N-CDs	74	200-5000	Fe(III)	[S3]
FNCDs	0.9	2–25	Fe(III)	[S4]
N/P-CDs	0.33	1−150	Fe(III)	[S5]
B/N−CDs	1.62	0-200	Fe(III)	[S6]
N-CDs	0.52	1-250	Fe(III)	[S7]
N-CDs	10.8	50-300	Fe(III)	[S8]
N-CDs	0.38	0.5–4	Fe(III)	[S9]
N-GQDs	0.09	1–1945	Fe(III), Hg(II)	[S10]
N-GQD	0.08	1-70	Fe(III)	[S11]
S-GQDs	0.0042	0-0.7	Fe(III)	[S12]
N-CNPs	0.20	1-500	Fe(III)	This work

N-CDs: nitrogen-doped carbon dots, FNCDs: fluorescent nitrogen-doped carbon dots, N/P-CDs: N, P codoped carbon dots, B/N−CDs: B,N codoped carbon dots, N-GQDs: nitrogen-doped graphene quantum dots, S-GQDs: sulfur-doped graphene quantum dots.

Table S3. The comparison between various fluorescent sensors based on CNPs or CDs toward GSH.

System	LOD (μM)	Linear range (μM)	Reference
Au(III)/CDC	2.02	0-150	[S13]
N,S-CQDs	6.7	0-50	[S14]
CQDs	0.943	1-50	[S15]
CDs-Br	0.14	0-34	[S16]
CDs–MnO2	0.6	1-200	[S17]
N-CNPs/Fe(III)	0.13	1~1000	This work

Au(III)/CDC: Au(III) decorated carbon dot cluster; N, S-CQDs: N, S co-doped carbon quantum dots; CQDs: carbon quantum dots; CDs-Br: bromoacetyl bromide functionalized CDs.

References

S1. Wang, C.; Hu, T.; Wen, Z.; Zhou, J.; Wang, X.; Wu, Q.; Wang, C. Concentration-dependent color tunability of nitrogen-doped carbon dots and their application for iron(III) detection and multicolor bioimaging. *J. Colloid. Interf. Sci.* **2018**, *521*, 33–41.

S2. Rao, L.; Tang, Y.; Li, Z.; Ding, X.; Liang, G.; Lu, H.; Yan, C.; Tang, K.; Yu, B. Efficient synthesis of highly fluorescent carbon dots by microreactor method and their application in Fe^{3+} ion detection.

Mater. Sci. Eng. C **2017**, *81*, 213–223.

S3. Chin, S.-F.; Tan, S.-C.; Pang, S.-C.; Ng, S.-M. Nitrogen doped carbon nanodots as fluorescent probes for selective detection and quantification of Ferric(III) ions. *Opt. Mater.* **2017**, *73*, 77-82.

S4. Atchudan, R.; Edison, T.N.J.I.; Aseer, K.R.; Perumal, S.; Karthik, N.; Lee, Y. R. Highly fluorescent nitrogen-doped carbon dots derived from *Phyllanthus acidus* utilized as a fluorescent probe for label-free selective detection of Fe^{3+} ions, live cell imaging and fluorescent ink. *Biosens. Bioelectron.* **2018**, *99*, 303–311.

S5. Shangguan, J.; Huang, J.; He, D.; He, X.; Wang, K.; Ye, R.; Yang, X.; Qing, T.; Tang, J. Highly Fe^{3+}-Selective Fluorescent Nanoprobe Based on Ultrabright N/P Codoped Carbon Dots and Its Application in Biological Samples. *Anal. Chem.* **2017**, *89*, 7477−7484.

S6. Liu, J.; Li, J.; Xu, L.; Qiao, Y.; Chen, J. Facile Synthesis of N, B-Doped Carbon Dots and Their Application for Multisensor and Cellular Imaging. *Ind. Eng. Chem. Res.* **2017**, *56*, 3905−3912.

S7. Song, Y.; Zhu, C.; Song, J.; Li, H.; Du, D.; Lin, Y. Drug-Derived Bright and Color-Tunable N-Doped Carbon Dots for Cell Imaging and Sensitive Detection of Fe^{3+} in Living Cells. *ACS Appl. Mater. Interfaces* **2017**, *9*, 7399−7405.

S8. Li, G.; Lv, N.; Bi, W.; Zhang, J.; Ni, J. Nitrogen-doped carbon dots as a fluorescence probe suitable for sensing Fe^{3+} under acidic conditions. *New J. Chem.* **2016**, *40*, 10213—10218.

S9. Liu, H.; Zhang, Y.; Liu, J.H.; Hou, P.; Zhou, J.; Huang, C.Z. Preparation of nitrogen-doped carbon dots with high quantum yield from Bombyx mori silk for Fe(III) ions detection. *RSC Adv*. **2017**, *7*, 50584–50590.

S10. Ju, J.; Chen, W. Synthesis of highly fluorescent nitrogen-doped graphene quantum dots for sensitive, label-free detection of Fe(III) in aqueous media. *Biosens. Bioelectron.* 2014, 58, 219–225.

S11. Xu, H.; Zhou, S.; Xiao, L.; Wang, H.; Lia, S.; Yuan, Q. Fabrication of a nitrogen-doped graphene quantum dot from MOF-derived porous carbon and its application for highly selective fluorescence detection of Fe^{3+}. *J. Mater. Chem. C* **2015**, *3*, 291–297.

S12. Li, S.; Li, Y.; Cao, J.; Zhu, J.; Fan, L.; Li, X. Sulfur-doped graphene quantum dots as a novel fluorescent probe for highly selective and sensitive detection of Fe^{3+}. *Anal. Chem.* **2014**, *86*, 10201−10207.

S13. Gu, J.; Hu, D.; Wang, W.; Zhang, Q.; Meng, Z.; Jia, X.; Xi, K. Carbon dot cluster as an efficient "off–on" fluorescent probe to detect Au(III) and glutathione. *Biosens. Bioelectron.* **2015**, *68*, 27–33.

S14. Song, Z.; Quan, F.; Xu, Y.; Liu, M.; Cui, L.; Liu, J. Multifunctional N,S co-doped carbon quantum dots with pH- and thermo-dependent switchable fluorescent properties and highly selective detection of glutathione. *Carbon* **2016**, 104, 169-178.

S15. Pan, J.; Zheng, Z.; Yang, J.; Wu, Y.; Lu, F.; Chen, Y.; Gao, W. A novel and sensitive fluorescence sensor for glutathione detection by controlling the surface passivation degree of carbon quantum dots. *Talanta* **2017**, *166*, 1–7.

S16. Yan, F.; Ye, Q.; Xu, J.; He, J.; Chen, L.; Zhou, X. Carbon dots-bromoacetyl bromide conjugates as fluorescence probe for the detection of glutathione over cysteine and homocysteine. *Sens. Actuators B Chem.* **2017**, *251*, 753–762.

S17. Xu, Y.; Chen, X.; Chai, R.; Xing, C.; Li, H.; Yin, X.-B. *Nanoscale* **2016**, *8*, 13414–13421.
